# Value of hospital administrative data linked to national cancer registry records to identify metastatic disease at time of primary diagnosis in colorectal cancer patients: a study using national data in England

**DOI:** 10.1186/s12885-025-13777-x

**Published:** 2025-03-06

**Authors:** Orouba Almilaji, Linda Sharples, Ajay Aggarwal, David Cromwell, Kieran Horgan, Michael Braun, Robert Arnott, Julie Nossiter, Angela Kuryba, Alexandra Lewin, Brian Rous, Thomas Cowling, Jan Van Der Meulen, Kate Walker

**Affiliations:** 1 Department of Health Service Research & Policy, Faculty of Public Health and Policy, London School of Hygiene & Tropical Medicine, London, UK; 2 Department of Oncology, Guy’s and St. Thomas’ NHS Foundation Trust, London, UK; 3 Clinical Effectiveness Unit, The Royal College of Surgeons of England, London, UK; 4 Department of Breast Surgery, Bexley Cancer Centre, St. James’s University Hospital, Leeds, UK; 5 The Christie NHS Foundation Trust, London, UK; 6 Green Templeton College, University of Oxford, Oxford, UK; 7 Cambridge University Hospitals NHS Foundation Trust, Cambridge, UK

**Keywords:** Metastasis, Colorectal cancer, Hospital administrative data, Cancer registry, SEARCHER project

## Abstract

**Background:**

Routinely collected data are increasingly being used for cancer research and health service evaluation. For both purposes, accurately identifying metastatic disease at diagnosis is essential. We developed an approach to identify metastatic disease at time of primary diagnosis according to national hospital administrative data (HAD) in patients identified with colorectal cancer (CRC) in the English national cancer registry (CR).

**Methods:**

A national cohort of CRC patients diagnosed between 2013 and 2018 in England identified in CR data were linked to HAD. Metastatic disease was assumed to be present at diagnosis according to HAD if at least one of a set of pre-specified diagnostic ICD-10 codes appeared in a record of a hospital admission between one month before and six months after CRC diagnosis date.

**Results:**

Of 186,236 patients, 40,421 (21.7%) had metastatic cancer according to HAD, 42,843 (23.0%) according to CR data, 49,827 (26.8%) according to either data source, and 33,437 (18.0%) according to both. Metastatic information was missing in CR data in 14,065 patients and 1,930 of these (13.7%) had metastatic cancer according to HAD. 1-year mortality was 59.3% (95%-CI: 58.8 − 59.8%) in patients with metastatic disease and 7.4% (7.2 − 7.5%) in patients without if HAD and CR data agreed. Mortality fell between these results if HAD and CR data disagreed. High mortality was seen in patients with missing metastatic data in the CR: 74.4% (72.4 − 76.3%) in patients with metastatic disease and 45.2% (44.3-46.1%) in patients without metastatic disease according to HAD.

**Conclusions:**

HAD should be linked to CR data to provide more accurate information on metastatic CRC at diagnosis including sites of metastasis. Linkage to HAD increased the number of patients identified with metastatic CRC by 14%, compared to CR data alone. Patients with metastatic disease at diagnosis in either data source had mortality outcomes expected for patients with metastatic cancer. CRC patients with missing metastasis data in CR data are likely to have metastatic disease and linkage to HAD provides important prognostic information.

**Supplementary Information:**

The online version contains supplementary material available at 10.1186/s12885-025-13777-x.

## Introduction

Patients with metastatic colorectal cancer (CRC) at the time of primary diagnosis have a poor prognosis with a 5-year mortality of around 90% compared to 10–30% in CRC patients without metastatic disease [1]. In England, nearly one quarter of patients with CRC have metastatic cancer at the time of diagnosis [2]. The most common metastatic sites are the liver, lung, and peritoneum [3]. Real-world data are increasingly being used for cancer research and service evaluation [4]. It is obvious that the value of this type of data to study patients with CRC depends on how well patients with metastatic disease at the time of diagnosis can be identified [5–7].

The English national cancer registry (CR) collects information on metastatic CRC at the time of diagnosis for the purposes of recording TNM stage at diagnosis [8]. However, metastatic disease is sometimes diagnosed during further investigations carried out a few weeks or months after the date of diagnosis which are not incorporated into TNM stage. In that case, this information about metastatic CRC– most likely already present at diagnosis, but not detected and recorded at that time– is often not included in the CR data. Another limitation of CR data is that they do not include information about the site of the metastatic disease [9].

In English hospital administrative data (HAD), ICD-10 diagnostic codes are recorded for each hospital episode in the National Health Service [10]. As a consequence, HAD have the potential to capture metastatic disease at the time of diagnosis as well as metastatic disease that is diagnosed slightly later. A further advantage of HAD is that diagnosis codes can indicate the site of the metastasis. On the other hand, it is well known that in many patients who were not admitted to hospital around the time of diagnosis, but for example managed in an outpatient setting, the recording of metastatic disease in HAD is incomplete.

The aim of this study was to investigate how well HAD and CR data linked at patient level identify patients with metastatic CRC around the time of primary diagnosis and the site of the metastatic disease. Firstly, we assessed the overall agreement between HAD and CR on the presence of metastatic CRC. Secondly, we investigated whether the agreement of the recording metastatic disease in HAD and CR correlates with key patient and tumour characteristics and the primary cancer treatment. Thirdly, we explored the timing of the recording of metastatic disease and the metastasis sites in HAD. Lastly, we compared cancer-specific mortality according to the agreement between HAD and CR on the presence of metastatic disease.

## Methods

### Data sources

This study used three national, routinely collected datasets linked at patient level. CR data from the National Disease Registration Service was used to identify all patients newly diagnosed with CRC in England between 1st January 2013 and 31st December 2018 [8, 11]. These CR data included the CRC diagnosis date and tumour characteristics, including information about cancer site, stage and grade [12].

The English Hospital Episode Statistics dataset includes records of all hospital episodes provided by the National Health Service in England [10]. We used these HAD records linked to the CR data at patient level to identify inpatient care episodes from 12 months before to 36 months after the CRC diagnosis to capture diagnoses recorded using the International classification of Diseases, 10th revision (ICD-10) codes [13] and procedures recorded using the Office of Population Censuses and Surveys Classification of Surgical Operations and Procedures, 4th revision (OPCS-4) codes [14]. The HAD records include patient characteristics, presence and site of CRC metastasis, presence of primary cancers other than CRC, and major resection. The first major CRC resection (Appendix 1) from one month before to one year after the CRC diagnosis was identified. The RCS Charlson comorbidity score was used to determine the number of comorbid conditions, not considering the presence of cancer [15]. The HAD records also include information about the date and cause of death provided by the Office for National Statistics for England and Wales.

The Systemic Anticancer Therapy (SACT) dataset was used to identify patients who received chemotherapy in the one month before to six months after CRC diagnosis. It includes information on date of receipt, regimen, cycle and drugs administered [16].

### Participants

**Fig. 1:**
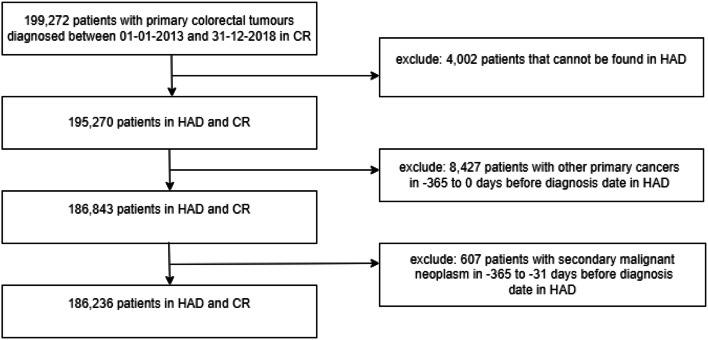
Flow chart presenting in- and exclusion criteria for the study cohort. HAD: hospital administrative data: CR: cancer registry

The analysis cohort included patients aged 18 years and above identified in CR data with a CRC diagnosis (ICD-10: C18-C20) whose record could be linked to at least one HAD record. Patients with a record of another primary cancer in CR or HAD (Appendix 2) in the 12 months before the CRC diagnosis were excluded to ensure that the metastatic disease detected in the CR or HAD data were most likely to be linked to CRC. Patients diagnosed with a metastatic malignant neoplasm (Appendix 3) according to HAD were excluded, if the first use of the code was more than one month before the CRC diagnosis. After these exclusions, 186,236 patients were included (Fig. 1).

### Metastasis information

A metastasis of CRC is defined as cancer that has spread to the peritoneal surface or at least one distant organ or lymph node [17]. Metastatic cancer detected at the time of diagnosis in CR is captured using TNM and Dukes classifications [18, 19]. We concluded that patients had metastatic disease according to CR data if stage at diagnosis was recorded as “M1”, “Dukes’ D”, or “stage 4”. If all three variables had missing information, metastatic cancer was treated as “missing”.

Patients were defined as having metastatic disease around the time of diagnosis according to HAD if at least one code from a set of diagnostic ICD-10 codes for metastatic cancer (Appendix 4) first appeared in a record of an admission between one month before and six months after the diagnosis date according to CR data (the “‘metastasis window”). Note that HAD information on metastatic disease could not be missing because patients either have at least one ICD-10 code for metastatic cancer in their hospital records during the metastasis window or none, and in the latter case they were considered not to have metastatic disease.

### Statistical methods

Analysis was descriptive with no formal hypothesis testing. We used Cohen’s kappa statistic to measure overall agreement between HAD and CR on the presence of metastatic cancer, excluding patients with missing CR data on the presence of metastases [20].

Cancer-specific mortality was estimated using non-parametric cumulative incidence considering non-cancer deaths as a competing event [21]. The time of CRC diagnosis according to CR data was time 0 and all patients were followed up for one year. 1-year cancer-specific mortality was compared according to the agreement between HAD and CR in order to assess the additional value of using HAD.

We carried out one sensitivity analysis, repeating the analyses with different lengths of the metastasis window and investigated its impact on the overall agreement of the identification of patients with metastatic CRC according to HAD and CR data.

Analyses were carried out in R (4.2.3) and RStudio (2023.03.0 Build 386).

## Results

### Overall agreement

Of the 186,236 CRC patients included, 40,421 (21.7%) had metastatic cancer according to HAD, 42,843 (23.0%) according to CR data, 49,827 (26.8%) according to either data source, and 33,437 patients had metastatic cancer according to both data sources (Cohen’s kappa 0.77; Table 1). Among 133,680 (124,274 + 9,406) patients without metastatic cancer according to HAD and complete metastasis information in CR, 9,406 (7.0%) had metastatic CRC according to CR data. Of the 14,065 (12,135 + 1,930) patients with missing metastasis information in CR data, 1,930 (13.7%) had metastatic cancer according to HAD.

**Table 1:** Agreement of data on metastatic disease according to hospital administrative data and cancer registry data (numbers)

		Hospital administrative data		All
		No metastasis	Metastasis	
Cancer registry data	No metastasis	124,274	5,054	129,328
	Metastasis	9,406	33,437	42,843
	Missing data	12,135	1,930	14,065
All		145,815	40,421	186,236

### Metastatic disease recording and patient and tumour characteristics

Compared to the 124,274 patients without metastatic cancer according to both data sources, the 47,897 (9,406 + 5,054 + 33,437) patients with metastatic cancer according to either data source were more likely to have their CRC diagnosed after an emergency admission and they were also more likely to have a higher grade, a larger primary CRC tumour (according to the T stage recorded in CR data), and lymph node involvement (according to the *N* stage recorded in CR data; Table 2).

**Table 2:** Patient and tumour characteristics comparing metastatic disease according to hospital administrative data and cancer registry data

	Hospital administrative data					
	No metastatic disease			Metastatic disease		
	Cancer registry data			Cancer registry data		
	No metastatic disease	Metastatic disease	Missing data	No metastatic disease	Metastatic disease	Missing data
	Number (%)	Number (%)	Number (%)	Number (%)	Number (%)	Number (%)
**All**	124,274	9,406	12,135	5,054	33,437	1,930
**Age (years)**						
18–74	73,545 (59.2)	4,421 (47.0)	3,605 (29.7)	3,182 (62.9)	20,541 (61.4)	817 (42.3)
75–84	36,212 (29.1)	3,009 (32.0)	3,408 (28.1)	1,411 (27.9)	8,970 (26.8)	614 (31.8)
≥ 85	14,517 (11.7)	1,976 (21.0)	5,122 (42.2)	461 (9.1)	3,926 (11.7)	499 (25.9)
**Number comorbidities**						
0	79,799 (64.2)	5,712 (60.7)	5,331 (43.9)	3,161 (62.5)	20,512 (61.3)	985 (51.0)
1	28,914 (23.3)	2,244 (23.9	3,287 (27.1)	1,279 (25.3)	8,345 (25.0)	512 (26.5)
≥2	15,561 (12.5)	1450 (15.4)	3517 (29.0)	614 (12.1)	4,580 (13.7)	433 (22.4)
**Diagnosed**						
Non-emergency	84,512 (83.2)	5,690 (74.5)	4,882 (51.3)	2,716 (67.3)	16,921 (62.0)	636 (42.0)
After emergency admission	17,012 (16.8)	1,944 (25.5)	4,636 (48.7)	1,319 (32.7)	10,352 (38.0)	879 (58.0)
*Missing (% of total)*	*22*,*750 (18.3)*	*1*,*772 (18.8)*	*2*,*617 (21.6)*	*1*,*019 (20.2)*	*6*,*164 (18.4)*	*415 (21.5)*
**Primary cancer site**						
Rectum	37,400 (30.1)	2,610 (27.7)	2,923 (24.1)	1,216 (24.1)	6,820 (20.4)	367 (19.0)
Colon	86,874 (69.9)	6,796 (72.3)	9,212 (75.9)	3,838 (75.9)	26,617 (79.6)	1,563 (81.0)
**Grade**						
Lower grade (1–2)	94,836 (86.5)	4,487 (74.4)	3,697 (85.8)	3,063 (70.9)	15,447 (73.7)	458 (69.3)
Higher grade (3–4)	14,855 (13.5)	1,545 (25.6)	613 (14.2)	1,258 (29.1)	5,521 (26.3)	203 (30.7)
*Missing (% of total)*	*14*,*583 (11.7)*	*3*,*374 (35.9)*	*7*,*825 (64.5)*	*733 (14.5)*	*12*,*469 (37.3)*	*1*,*269 (65.8)*
**T stage**						
Smaller tumour (T1-T2)	35,469 (29.4)	471 (6.8)	684 (46.7)	464 (9.5)	957 (4.5)	12 (8.7)
Larger tumour (T3-T4)	85,106 (70.6)	6,450 (93.2)	781 (53.3)	4,400 (90.5)	20,153 (95.5)	126 (91.3)
*Missing (% of total)*	*3*,*699 (3.0)*	*2*,*485 (26.4)*	*10*,*670 (87.9)*	*190 (3.8)*	*12*,*327 (36.9)*	*1*,*792 (92.8)*
**N stage**						
No lymph nodes (N0)	73,002 (60.5)	1,456 (21.0)	545 (54.4)	1,493 (30.5)	3,623(16.9)	28 (25.7)
Lymph nodes (N1-N2)	47,579 (39.5)	5,493 (79.0)	456 (45.6)	3,404 (69.5)	17,868 (83.1)	81 (74.3)
*Missing (% of total)*	*3*,*693 (3.0)*	*2*,*457 (26.1)*	*11*,*134 (91.8)*	*157 (3.1)*	*11*,*946 (35.7)*	*1*,*821 (94.4)*

On average, patients with missing metastasis information in the CR were older, had more comorbid conditions, and were more frequently diagnosed after an emergency admission, compared to patients with non-missing CR data. Moreover, patients with missing metastasis information in the CR were more likely to have missing data on T- and N-stage, compared to patients with non-missing CR data. Conversely, patients with metastatic CRC according to CR data were more likely to have missing information on cancer grade, T- and N-stage, compared to patients with non-metastatic CRC in CR data.

The agreement on presence of metastatic disease between data sources did not vary widely between NHS hospital organisations (Appendix 5). For 90% of the hospital organisations, we observed “full agreement” between HAD and CR (i.e., presence or absence of metastatic disease according to both sources among patients with non-missing metastasis information in the CR) for between 77.1% and 90.1% of patients.

### Metastatic disease and primary treatment

Of the 124,274 patients without metastatic disease according to both data sources, 63,932 (51.4%) had a major resection without chemotherapy as their primary treatment, 31,704 (25.5%) had a major resection with chemotherapy, and 3,702 (3.0%) had chemotherapy without a major resection (Table 3). Of the 33,437 patients with metastatic cancer according to both data sources, 3,043 (9.1%) had a major resection without chemotherapy, 6,143 (18.4%) had a major resection with chemotherapy and 10,398 (31.1%) had chemotherapy without a major resection.

**Table 3:** Major resection and chemotherapy comparing metastatic disease according to hospital administrative data and cancer registry data

		Hospital administrative data					
		No metastatic disease			Metastatic disease		
		Cancer registry data			Cancer registry data		
		No metastatic disease	Metastatic disease	Missing data	No metastatic disease	Metastatic disease	Missing data
		Number (%)	Number (%)	Number (%)	Number (%)	Number (%)	Number (%)
**All**		124,274 (100)	9,406 (100)	12,135 (100)	5,054 (100)	33,437 (100)	1,930 (100)
**Major resection**	**Chemotherapy**						
No	No	24,936 (20.1)	4,906 (52.2)	10,479 (86.4)	828 (16.4)	13,853 (41.4)	1,443 (74.8)
No	Yes	3,702 (3.0)	1,396 (14.8)	316 (2.6)	642 (12.7)	10,398 (31.1)	305 (15.8)
Yes	No	63,932 (51.4)	1,431 (15.2)	1,057 (8.7)	1,622 (32.1)	3,043 (9.1)	106 (5.5)
Yes	Yes	31,704 (25.5)	1,673 (17.8)	283 (2.3)	1,962 (38.8)	6,143 (18.4)	76 (3.9)

Patients for whom the data sources disagreed on metastatic cancer tended to have different primary treatments. 4,226 (83.6%) of the 5,054 patients with metastatic cancer according to HAD but not CR data underwent major resection or chemotherapy, whereas only 4,500 (47.8%) of the 9,406 patients with metastatic cancer according to CR data but not according to HAD underwent major resection or chemotherapy. 24,936 of the 124,274 patients with non-metastatic disease according to both the CR and HAD data were not treated with a major resection and/or chemotherapy.

Patients with missing information in the CR about metastatic disease had the lowest rates of major resection and chemotherapy. Of these patients, 12,135 did not have metastatic disease according to HAD and only 1,656 (13.6%) had a major resection, chemotherapy or both, whereas 1,930 patients had metastatic disease according to HAD and only 487 (25.2%) had a major resection, chemotherapy or both.

### Timing of recording of metastatic disease in hospital administrative data

**Fig. 2:**
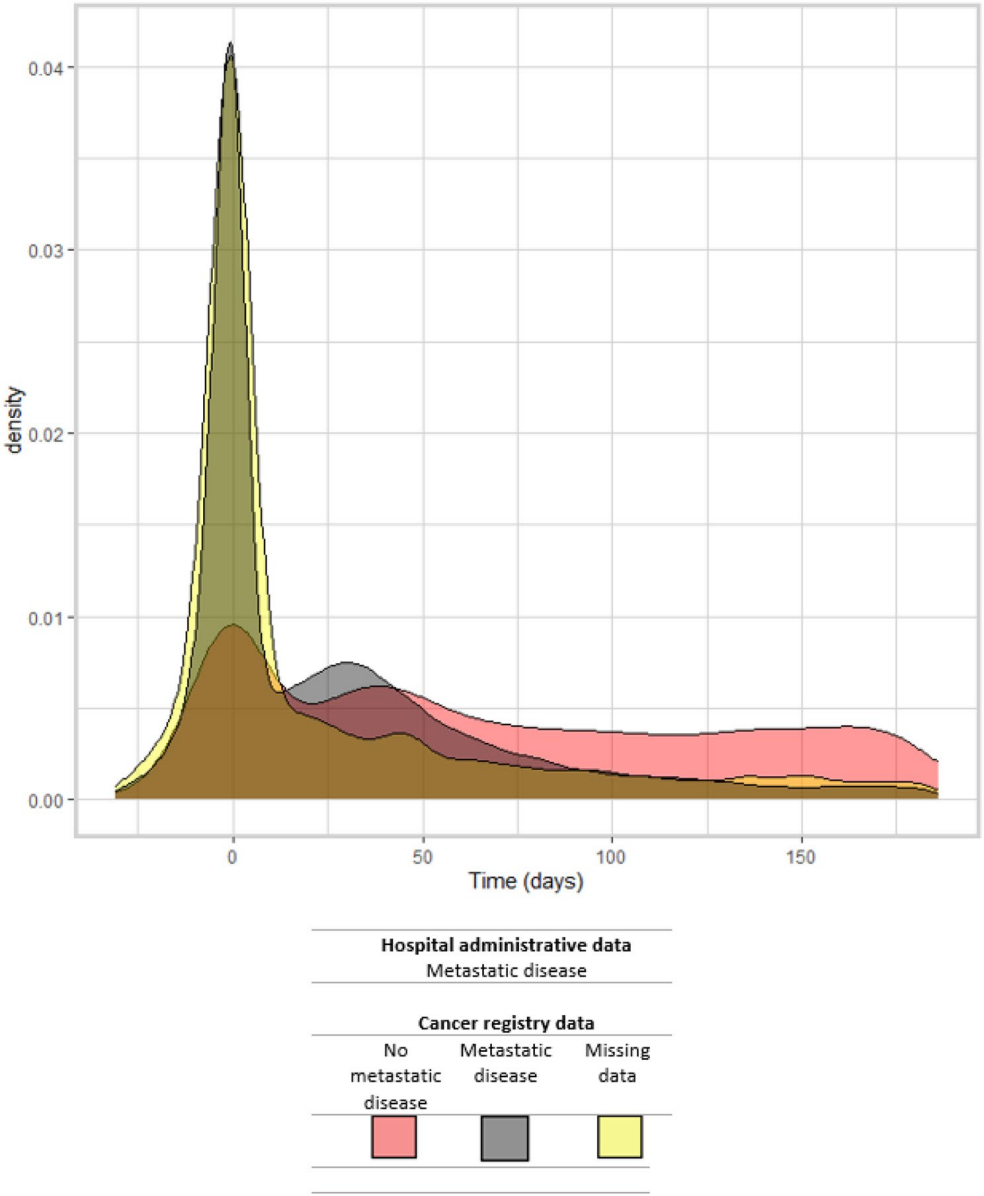
Time from CRC diagnosis to earliest metastasis in 40,421 patients with metastatic disease in hospital administrative data

Of the 40,421 patients with metastatic cancer according to HAD, 25,312 (62.6%) had a metastasis recorded in the period from one month before to one month after the CRC diagnosis date, with the largest peak centred on the date of CRC diagnosis and a smaller second peak around 4 to 6 weeks later (Fig. 2). The shape of the distribution depended on whether or not metastatic disease was recorded in CR data. 22,191 of the 33,437 (66.4%) patients who had metastatic cancer according to CR data had metastatic cancer recorded in HAD within 1 month of the CRC diagnosis date, whilst the corresponding figures were are 1,711 of the 5,054 patients (33.9%) who did not have metastatic cancer according to CR data, and 1,428 of the 1903 patients (73.9%) with missing data on metastatic cancer in CR data.

### Metastatic site according to hospital administrative data

The liver was the most frequently recorded metastasis site, followed by lung, and peritoneum (Appendix 6). Around half of patients with metastatic disease according to HAD had more than one metastatic site (21,418 / 40,421, 53.0%). The most common metastatic sites were the liver only (31.2%), followed by the liver and lung (16.8%), the peritoneum only (8.0%), and the peritoneum and liver (6.8%).

### Cancer-specific mortality

**Fig. 3:**
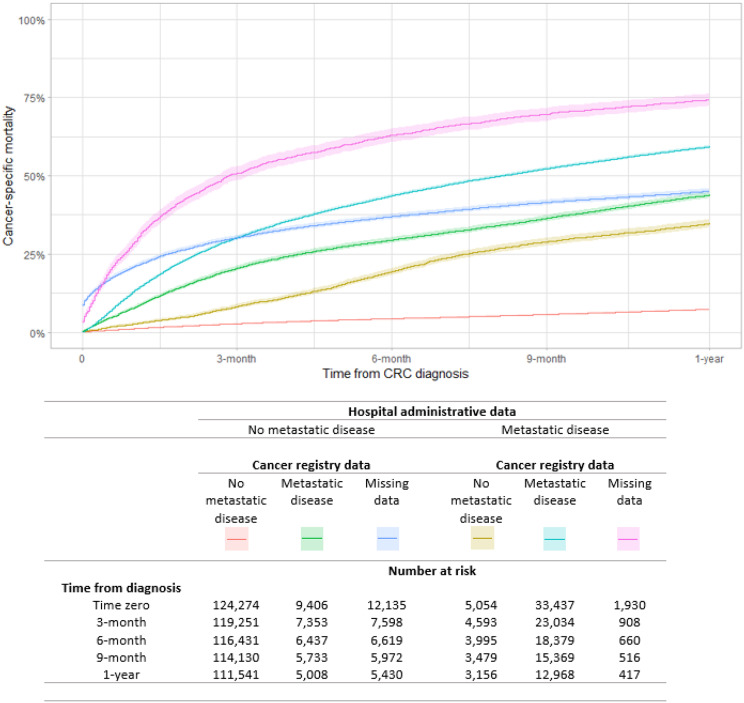
Cancer-specific cumulative incidence (time 0 is the date of CRC diagnosis, event of interest is cancer death, competing risk: other causes of death)

The one-year cancer-specific mortality of the 33,437 patients with metastatic disease according to both HAD and CR data was 59.3% (95% CI:58.8–59.8%) compared to 7.4% (95%CI: 7.2–7.5%) in the 124,274 patients without metastatic disease according to both HAD and CR data (Fig. 3). The one-year cancer-specific mortality fell below 59.3% for the patients for whom HAD and CR data disagreed on the presence of metastatic cancer: it was 34.9% (95% CI: 33.6–36.2%) in the 5,054 patients with metastatic disease according to HAD but not CR data and 43.5% (95% CI: 42.9–44.9%) in the 9,406 patients with metastatic disease according to CR data but not HAD. The 1-year cancer-specific mortality was 45.2% (95% CI: 44.3–46.1%) in the 12,135 patients without metastatic disease according to HAD but with missing metastatic disease in CR data, and 74.4% (95% CI: 72.4–76.3%) in the 1,930 patients with metastatic disease according to HAD but missing metastasis information in the CR, which was by far the worst 1-year outcome of all comparison groups.

### Sensitivity analysis

In the sensitivity analysis, we first changed the end date of the “metastasis window” from six months to three months after the CRC diagnosis date. This decreased the number of patients identified with metastatic CRC according to HAD from 40,421 to 35,520 (decrease of 12.1%). Changing the end date of the metastasis window to only one month after the CRC diagnosis date, the number of patients with metastatic CRC decreased further to 25,566 (decrease of 36.7%). The impact of shortening the metastasis window was largest in the patients identified with metastatic disease in HAD but not in CR: a decrease from 5,054 to 3,272 (decrease of 35.3%) with an end date of the metastasis window of three months after the CRC diagnosis and to 1,732 (decrease of 65.7%) with an end date of one month after the CR diagnosis.

## Discussion

Linked HAD and CR data detected metastatic cancer in 26.8% of patients around the time of the primary CRC diagnosis. This percentage is about 30% higher than the 20% that is typically quoted for newly diagnosed CRC patients in England [2]. Metastatic cancer was detected in 23.0% of the patients when only CR data were used, and in 21.7% when only HAD data were used. In other words, one in seven patients with metastatic disease was not identified in CR data alone and one in five was not identified in HAD alone. Patients with metastatic disease according to either data source had patient and tumour characteristics that can be expected in patients with metastatic CRC around the time of diagnosis.

The treatment patterns varied between the agreement groups. The highest percentage of patients not receiving any cancer treatment was observed when CR data on metastatic disease was missing, irrespective of whether or not patients had metastatic disease according to HAD. It is also important to note that the percentage not having a major resection or chemotherapy was much higher in those patients who had metastatic CRC according to CR data but not according to HAD than in those who had metastatic CRC according to HAD but not according to CR. All these results demonstrate that both missing information on metastatic disease in the CR and the pattern of disagreement between HAD and CR reflect specific clinical factors and circumstances.

Cancer-specific mortality also varied according to the pattern of agreement on metastasis between the data sources. Patients with metastatic disease according to HAD but with missing metastasis data in the CR had the highest 1-year cancer-specific mortality of all patients.

Lastly, in contrast to CR data, HAD identified the sites of the metastases. For example, HAD demonstrated that the metastasis sites were identified in the liver, peritoneum and lung– either isolated or in combination– in about 70% of the patients, which is broadly consistent with results reported the recent literature [3].

To understand the pattern of agreement between HAD and CR in identifying metastatic disease around the time of a CRC diagnosis, we must firstly consider how clinical information “flows” into each data source. The English National Disease Registration Service focuses its dedicated data capture on information that is available *at the time of diagnosis*. In contrast, the Hospital Episode Statistics, the HAD source in the English National Health Services, routinely collates a record of all hospital episodes (in other words, any time before, around and after a cancer diagnosis). Hence, HAD data are a more appropriate data source to follow up cancer patients over time than CR data. As a consequence, the HAD used in our study captures clinical cancer staging information that may already be available *before the cancer diagnosis date* as well as *later in the patient pathway*. We saw a second peak in the distribution of the timing of metastatic recording in HAD at around 4 to 6 weeks after the date of diagnosis, which fits with the timing of results being available from further diagnostic investigations.

The typical treatment pathways for some groups of CRC patients will influence the detection of metastatic disease. For example, we found that the percentage of patients with metastatic disease according to CR but not according to HAD who do not undergo a major resection or chemotherapy is relatively high (52.2%). This could be a group of patients with metastatic disease detected at time of diagnosis who underwent little further staging investigation or active cancer treatment. These patients are likely to have had fewer admissions to hospital and are therefore less likely to have their diagnoses captured in HAD. In contrast, only 16.4% of patients with metastasis according to HAD but not according to CR data had neither major resection nor chemotherapy. These are likely to be patients with metastatic disease who underwent active treatment for their CRC but whose metastatic disease was erroneously missed when their cancer diagnosis was recorded in the national cancer registry. A sizeable proportion (20.1%) of patients without metastatic disease according to both the CR and HAD data did not have a major resection or chemotherapy. This may be due to inclusion of patients with a rectal tumour being treated with radiotherapy as an alternative to surgery, or patients having minor procedures like endoscopic resection or endoscopic polypectomy, patients not receiving active treatment because of their age or comorbidities.

Our findings demonstrate the importance of linking HAD to CR data to improve the completeness of the identification of newly diagnosed CRC patients with metastatic disease. However, studies using these linked data also should consider distinguishing these separate groups of CRC patients with metastatic disease according to the pattern of agreement between the two data sources given that they differ in terms of patient characteristics, treatment patterns and outcomes.

Some studies aim to investigate the prognosis of patients whose metastatic disease was known *at the time of diagnosis.* For these studies, only patients with metastatic disease at diagnosis identified in CR data or identified in HAD based on records of care episodes that occurred *up to the date of diagnosis* should be considered to have metastatic disease. Other studies may investigate the effectiveness of particular treatments between groups, with adjustment for differences in the prevalence of metastatic disease *present at the date of diagnosis*, irrespective of whether the patients with metastatic disease were *identified up to the date of diagnosis.* For these studies, all recorded metastatic disease, including metastasis in hospital episodes in HAD a*fter the date of diagnosis*, should be included as long as it can be assumed that the metastatic disease was already present at the time of diagnosis. In summary, there is a distinction between studies that need to identify patients with metastatic disease *detected* at the time of the CRC diagnosis and studies that need to identify patients with metastatic disease *present* at the time of diagnosis.

Our study demonstrates that the missing metastasis information in CR data is highly informative or, in other words, the information on metastatic disease cannot be assumed to be missing at random. Patients with missing CR data were least likely to have major resection or chemotherapy and they had the highest mortality. In this case, the recommended approach, least likely to generate biased results, is to include these patients as a separate group rather than to exclude them from the analysis or to use approaches to impute that missing data based on the patients’ other known characteristics [22]. It has been argued that the validity of including patients with missing information as a separate group, depends on the extent to which one can expect that the mechanism that is responsible for the pattern of missingness is “transportable” to other locations or future settings and this aspect will need to be judged for each study on a case-by-case basis [23].

Our study is the first to compare the information on metastatic disease in CRC patients according to HAD and CR data. We used national datasets and our findings and recommendations are therefore likely to be generalisable to CRC patients in other high-income countries with similar HAD and CR sources. However, 2% of newly diagnosed CRC patients identified in CR data could not be linked to HAD and were therefore not included in the study. Excluding a relatively small proportion of patients is unlikely to have had a substantial impact on our findings. Whilst the availability, completeness and accuracy of databases differ between countries, the added value of linking routine administrative data (such as HAD or healthcare claims data) to disease-specific data (such as CR data), in order to better identify the nature and details of clinical episodes (such as metastasis and site of metastasis) is relevant to any country with similar data sources. Further, the definitions of metastasis, TNM staging information and ICD-10 codes that are used in this study are international. In practice, missing information on the staging of CRC at diagnosis due to, for example, patient frailty, high levels of co-morbidity and older age, is a common problem, and has been reported for cancer registries in the USA, Japan, Canada and Ireland [24–26].

The HAD used in our study included patients who were admitted to hospital, including day-case admissions. A key question that remains is to what extent records of outpatient or emergency department visit would improve the capture of metastatic disease. In our context, recording of diagnoses and procedures in outpatient and emergency department visit data is often incomplete, and therefore we cannot address this question [27].

## Further work

This study has important implications for research and service evaluation for all cancers, and similar validation work should be carried out for other primary cancer sites. Our methods should be integrated into the data production processes of national cancer registries to provide national information on metastases present around the time of diagnosis.

We investigated how well HAD and CR data linked at patient level identified patients with metastatic CRC at the time of diagnosis. However, there is also an important need to carry out similar work assessing how well these linked data can be used to detect cancer disease progression, for example by identifying patients who did not have metastatic disease at the time of diagnosis but who develop metastatic disease later in the course of their cancer [28]. The findings of our current study, only investigating the presence of metastatic cancer at the time of diagnosis, suggest that HAD may be a valuable source of this much-needed follow-up information.

## Conclusions

Routinely linking HAD to CR data leads to more complete identification of patients with metastatic disease at the time of their CRC diagnosis. However, patients with metastatic disease with different patterns of agreement between the two data sources should be considered separately as they may differ in terms of patient characteristics, treatment patterns, and outcomes. Also, patients with missing data on metastatic disease in CR data form a highly selected group and analytical approaches should be adequate for handling observations with a pattern of missingness that is not at random.

## Patient and public involvement (PPI)

This study was carried out with patients and representatives of cancer charities on the Study Steering Committee of the research project, led by the research study co-applicant, Robert Arnott, consulted on all aspects of the study.

## Patient perspective on the research findings

This research has implications for cancer research using routinely collected health data. It provides insights that may enhance the quality of future cancer studies using this type of data. The results demonstrate the importance of methods to improve the completeness of staging information, given that patients with missing information have poor outcomes. It is important for patients to understand where CR data and HAD comes from, where colorectal cancer spreads, to and the outcomes stratified by the site of metastases and how valid analyses of accurate and complete routinely collected healthcare data can contribute to the improvement of available treatment for future patients.

## Electronic supplementary material

Below is the link to the electronic supplementary material.

Supplementary Material 1

## Data Availability

Data available from NHS England’s Data Access Request Service (DARS).
