# Supplementary material for: Value of hospital administrative data linked to national cancer registry records to identify metastatic disease at time of primary diagnosis in colorectal cancer patients: a study using national data in England
Source: BMC Cancer. 2025 Mar 6;25:407. doi: 10.1186/s12885-025-13777-x (PMC11887144; doi:10.1186/s12885-025-13777-x)
Supplement: Supplementary file 1 — Supplementary Material 1 [file 12885_2025_13777_MOESM1_ESM.docx]

## Appendices

### Appendix 1: OPCS-4 codes to identify major resection in Hospital Administrative data

- H04 Total excision of colon and rectum
- H05 Total excision of colon
- H06 Extended excision of right hemicolon
- H07 Other excision of right hemicolon
- H08 Excision of transverse colon
- H09 Excision of left hemicolon
- H10 Excision of sigmoid colon
- H11 Other excision of colon
- H29 Subtotal excision of colon and rectum (Clean contaminated
- H33 Excision of rectum (Clean contaminated
- H411 Rectosigmoidectomy and peranal anastomosis
- H414 Peranal mucosal proceduretectomy and endoanal anastomosis
- H471 Excision of sphincter of anus
- H479 Unspecified excision of anus
- X141 Total exenteration of
- X142 Anterior exenteration of pelvis
- X143 Posterior exenteration of pelvis
- X148 Other specified clearance of
- X149 unspecified clearance of pelvis
- H404 Trans-sphincteric anastomosis of colon to anus

### Appendix 2: ICD-10 codes to identify primary cancers of another site in Hospital Administrative data

- C00, C01, C02, C03, C04, C05, C06, C07, C08, C09, C10, C11, C12, C13, C14 lip, oral cavity
- C15 oesophagus
- C16 stomach
- C17 small intestine
- C21 anal canal
- C22 liver &bile duct
- C23 gallbladder
- C24 biliary tract
- C25 pancreas
- C261 spleen
- C30, C31, C32, C33, C34, C37, C38, C39 respiratory and intrathoracic organs
- C40, C41 bone
- C43 melanoma of skin
- C45, C46, C47, C48, C49 mesothelial and soft tissue
- C50 breast
- C51, C52, C53, C54, C55, C56, C57, C58 female genital organs
- C60, C61, C62, C63 male genital organs
- C64, C65, C66, C67, C68 urinary tract
- C69, C70, C71, C72 eye, brain and other parts of central nervous system
- C73, C74, C75 thyroid and other endocrine glands
- C760 Head, face and neck
- C761 Thorax
- C762 Abdomen
- C763 Pelvis
- C764 upper limb
- C765 lower limb
- C767 other specified ill-defined sites (back, flank, trunk)
- C768 overlapping lesion (abdominal pelvic)
- C802 Malignant neoplasm associated with transplanted organs
- C81, C82, C83, C84, C85, C86, C88, C90, C91, C92, C93, C94, C95, C96 lymphoid, hematopoietic and related tissue
- C97 independent (primary) multiple sites (we want patient with CRC cancer only)

ICD-10 codes for primary cancer (not considered for exclusion because it is (or may be) in the same site, or very common and its occurrence and treatment will not affect the bowel cancer treatment)

- C18, C19, C20 colon, rectosigmoid junction, and rectum
- C260 unspecified intestinal tract
- C268 Overlapping lesion of digestive system
- C269 ill-defined sites within the digestive system
- C4A Merkel cell carcinoma (skin)
- C44 Other and unspecified malignant neoplasm of skin
- C801 Malignant (primary) neoplasm, unspecified
- C800 Disseminated malignant neoplasm, unspecified

Note: Cancer registry data was provided for 799,985 patients with primary colorectal, breast, prostate, and/or upper-gastrointestinal tumours diagnosed between 01-01-2013 and 31-12-2018 in CR. Before linking with Hospital administrative data, 600713 patients with breast, prostate, and upper-gastrointestinal tumours in CR were excluded.

### Appendix 3: ICD-10 codes to identify secondary cancers in Hospital Administrative data

- C77 Secondary and unspecified malignant neoplasm of lymph nodes
- C78 Secondary malignant neoplasm of respiratory and digestive organs
- C79 Secondary malignant neoplasm of other and unspecified sites
- C800 Disseminated malignant neoplasm, unspecified

### Appendix 4: ICD-10 codes to identify distant metastases in Hospital Administrative data

- C770 head, neck, and face lymph nodes
- C771 intrathoracic lymph nodes
- C773 axillary and upper limb nodes
- C774 inguinal and lower limb nodes
- C780, C781, C782, C783 respiratory organs
- C784 small intestine
- C786 peritoneum
- C787 liver metastasis
- C790, C791 urinary organ
- C792 skin
- C793, C794 brain and cerebral meninges
- C795 bone
- C796 ovary
- C797 adrenal gland
- C7981 breast
- C7982 genital organs
- C7989 secondary malignant neoplasm of other specified sites (intra-abdominal organs, heart, chest wall)

### Appendix 5: Variability in the metastasis information among NHS Trusts.

Number of NHS trusts is 163. Number of patients in these NHS trusts is 185323 (99.5% of the cohort). Number of patients in each of these trusts >50. Bars are sorted in descending order per the total number of patients.


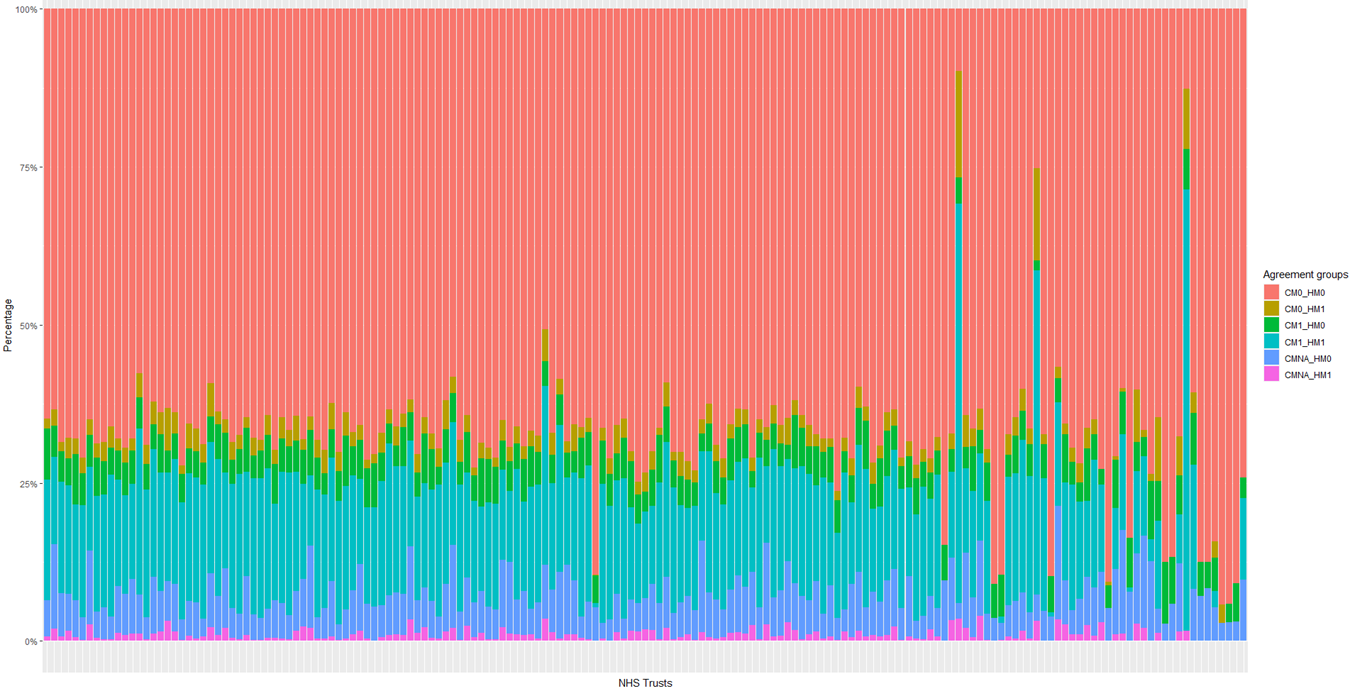


### Appendix 6: Sites of metastasis in 40,421 patients with metastatic disease according to hospital administrative data.

| **Metastasis site** | **Number (%).** |
| --- | --- |
|  |  |
| **All patients** | 40,421 |
|  |  |
| Liver only | 12,592 (31.2%) |
| Lung, liver | 6,805 (16.8%) |
| Peritoneum only | 3,235 (8.0%) |
| Peritoneum, liver | 2,759 (6.8%) |
| Lung, peritoneum, liver | 1,676 (4.2%) |
| Lung only | 1,495 (3.7%) |
| Lung, liver, bone | 965 (2.4%) |
| Liver, bone | 742(1.8%) |
| Other sites /combinations of metastasis sites | 10,152 (25.1%) |
